# Supplementary material for: Discovery of common chemical exposures across three continents using silicone wristbands
Source: R Soc Open Sci. 2019 Feb 6;6(2):181836. doi: 10.1098/rsos.181836 (PMC6408398; doi:10.1098/rsos.181836)
Supplement: Supplemental Material 1 [file rsos181836supp1.pdf]

Supplemental Material

Discovery of common chemical exposures across three continents using silicone wristbands

Holly M. Dixon[1]; Georgina Armstrong[2]; Michael Barton[1]; Alan J. Bergmann[1]; Melissa Bondy[2]; Mary L. Halbleib[3]; Winifred Hamilton[4]; Erin Haynes[5]; Julie Herbstman[6]; Peter Hoffman[1]; Paul Jepson[7]; Molly L. Kile[8]; Laurel Kincl[8]; Paul J. Laurienti[9]; Paula North[10]; L. Blair Paulik[1]; Joe Petrosino[11]; Gary L. Points III[1]; Carolyn M. Poutasse[1]; Diana Rohlman[8]; Richard P. Scott[1]; Brian Smith[1]; Lane G. Tidwell[1]; Cheryl Walker[12]; Katrina M. Waters[13]; Kim A. Anderson[1*]

[1]Food Safety and Environmental Stewardship Program, Environmental and Molecular Toxicology, Oregon State University, Corvallis, Oregon, USA
[2]Department of Medicine, Section of Epidemiology and Population Sciences, Baylor College of Medicine, Houston, Texas, USA
[3]Department of Crop and Soil Science, Oregon State University, Corvallis, Oregon, USA
[4]Department of Medicine, Environmental Health Section, Baylor College of Medicine, Houston, Texas, USA
[5]College of Medicine, Department of Environmental Health, University of Cincinnati, Cincinnati, Ohio, USA
[6]Columbia Center for Children's Environmental Health, Department of Environmental Health Sciences, Mailman School of Public Health, Columbia University, New York, New York, USA
[7]Integrated Plant Protection Center, Oregon State University, Corvallis, Oregon, USA
[8]College of Public Health and Human Sciences, Oregon State University, Corvallis, Oregon, USA
[9]Department of Radiology, Wake Forest School of Medicine, Winston-Salem, North Carolina, USA
[10]Department of Pathology, Medical College of Wisconsin, Milwaukee, Wisconsin, USA
[11]Department of Molecular Virology and Microbiology, Baylor College of Medicine, Houston, Texas, USA
[12]Department of Medicine, Center for Precision Environmental Health, Baylor College of Medicine, Houston, Texas, USA
[13]Biological Sciences Division, Pacific Northwest National Laboratory, Richland, Washington, USA

Note: *See **Supplemental Material - Data** for the dataset.* Chemicals detected are indicated with a 1 and chemicals not detected are indicated with a 0.

Table of Contents

Page	Content
S2	**Table S1.** GC-MS control parameters as previously reported in Bergmann et al. 2018.
S3	**Table S2.** Venn diagram table for all detected chemicals in three unique groups of wristbands, corresponding to Figure 3A.
S4	**Table S3.** Venn diagram table for the 30% most commonly detected chemicals in three unique groups of wristbands, corresponding to Figure 3B.
S5	**Table S4.** Venn diagram table for all detected chemicals in five unique groups of wristbands, corresponding to Figure 3C.
S6	**Table S5.** Venn diagram table for the 30% most commonly detected chemicals in five unique groups of wristbands, corresponding to Figure 3D.
S7-8	**Table S6.** Tukey-Kramer HSD results for different regions, represented by (A) connecting letters of significance and (B) p-values.
S9	**Table S7.** Tukey-Kramer HSD results for rural and urban groups within different continents, represented by (A) connecting letters of significance and (B) p-values.
S10-11	**Table S8.** Tukey-Kramer HSD results for different age groups, represented by (A) connecting letters of significance and (B) p-values.
S12	**Table S9.** Tukey-Kramer HSD results for males and females within different continents, represented by (A) connecting letters of significance and (B) p-values.
S13	**Figure S1.** Chemical pairs detected together ≥20 times in wristband samples.
S14	**Figure S2.** The PCA shown in Figure 5, but the symbol color here indicates the presence (orange) and absence (pink) of bis(2-ethylhexyl) phthalate.
S15	**Figure S3.** Number of chemical detections compared with wristband deployment time for all wristbands.

Table S1. GC-MS control parameters as previously reported in Bergmann et al. 2018 [1].

**GC-MS Settings**		
Injection volume (µL)	1	
Mode	Pulsed Splitless	
Heater (°C)	265	
Pressure (psi)	RTL*	
Total flow (mL/min)	RTL*	
Septum purge flow (mL/min)	2	
System Carrier Gas	Helium	
Injection pulse pressure (psi)	25 until 0.5 min	
Purge flow to split vent (mL/min)	20 at 0.45 min	
Transfer line (°C)	300	
Column flow (mL/min)	RTL*	
Average velocity (cm/s)	RTL*	
**Temperature Program**		
**Time (min)**	**Ramp (°C/min)**	**Temp (°C)**
0	0	70
2	25	150
5.2	3	200
21.9	8	280
31.9	0	280
46.9	40	310
47.7	0	310
50.7	20	325
51.45	0	325
53.37	End Method	
**Mass Spec Settings**		
Source temp(°C)	300	
Quad temp (°C)	150	
**Column Settings & Specifications**		
Column Type	Agilent DB-5MS	
Length (m)	30	
Diameter (µm)	250	
Film thickness (µm)	0.25	

***RTL: retention time lock, pressure and associated parameters are adjusted based on instrument operating conditions

Table S2. Venn diagram table for all detected chemicals in three unique groups of wristbands, corresponding to Figure 3A. Black dots with a blue background represent the wristband groups included for each intersection of the Venn diagram.

Intersections	Wristband Groups: North America	Wristband Groups: South America	Wristband Groups: Africa	Chemicals in Common: Number	Chemicals in Common: Names
One	•			68	1-naphthylamine, 2,3,6-trimethylphenol, 2,4-dimethylaniline, 2-chlorosyringaldehyde, 2-isopropylphenol, 2-sec-butylphenol, 4-tert-butylphenol, 9-methylanthracene, PBB 1, PBDE 51, PBDE 88, PBDE 100, PBDE 119, PBDE 47, PBDE 49, PBDE 99, PCB 102, PCB 88, PCB 93, PCB 95, PCB 98, TCEP, acenaphthene, alpha-chlordane, benzo[a]fluoren-11-one, benzo[a]fluorene, benzo[ghi]perylene, benzyl cinnamate, bifenthrin, bioallethrin S-cyclopentenyl isomer, bisphenol A, carvone, cinnamal, citral B, cyclopentadecanone, cyfluthrin I, cyfluthrin II, cyfluthrin III, cypermethrin-3, cyphenothrin trans-, d-(cis-trans)-phenothrin-I, drometrizole, ethofenprox, fipronil, desulfinyl-, fipronil-sulfone, gamma-chlordane, isobornyl thiocyanoacetate, jasmolin II, kinoprene, methoprene II, methyleugenol, p-nitrotoluene, pentachloroanisole, phenoxyacetic acid, phthalimide, promecarb, pyriproxyfen quinolone, rabenzazole, terbucarb, tetrachlorvinphos, theobromine, tilt, trans-nonachlor, tri-p-tolyl phosphate, tricresylphosphate meta-, tricresylphosphate ortho-, triphenylene
		•		22	1,5-dimethylnaphthalene, 1-methylpyrene, 2,4'-DDT, 2,6-dimethoxyphenol, 2-ethylnaphthalene, 3,5-dichloroaniline, 4,4'-DDE, 4-chlorophenol, 4-methylphenol, 9-fluorenone, bendiocarb, benzo[a]pyrene, bisphenol AF, butachlor, cyclopenta[cd]pyrene, dichlorvos, diphenylamine, pirimiphos-methyl, tebuconazole, tetramethrin II, traseolide, trifloxystrobin
			•	6	1-hydroxynaphthalene, 2,3,5-trimethylphenol, musk xylene, profenofos metabolite (4-bromo-2-chlorophenol), propoxur, tetramethrin I
Two		•	•	0	
	•		•	5	benzyl alcohol, celestolide, cyhalothrin (gamma), cyhalothrin I (lambda), triethyl phosphate
	•	•		54	1,2-dimethylnaphthalene, 1,6-dimethylnaphthalene, 1-methylphenanthrene, 2,3-dimethylanthracene, 2,4-di-tert-butylphenol, 2,4-dichlorophenol, 2-methoxy-4-methylphenol, 2-methylnaphthalene, 2-methylphenanthrene, 3,6-dimethylphenanthrene, 3-chloroaniline, 3-tert-butylphenol, 4,4'-DDD, 4,4'-DDT, 4-chloro-3,5-dimethylphenol, 4-isopropylphenol, TCPP, TPP, a-ionone, acenaphthylene, b-citronellol, b-ionone, benz[a]anthracene, benzo[b]fluorene, biphenyl, cashmeran, chlorpyrifos, chrysene, citral A, cypermethrin-2, cyphenothrin, cis-diamyl phthalate, dicyclohexyl phthalate, dimethyl phthalate, diuron metabolite (3,4-dichlorophenyl isocyanate), farnesol IV, fipronil, fluoranthene, geraniol, guaiacol, isoeugenol, lyral, malathion, naphthalene, nicotine, o-phenylphenol, permethrin, permethrin II, phenanthrene, piperonyl butoxide, promecarb artifact (5-isopropyl-3-methylphenol), retene, tributyl phosphate, tris(2-ethylhexyl) phosphate
Three	•	•	•	36	1-methylnaphthalene, 2,6-dimethylnaphthalene, 4-chlorophenyl isocyanate, amyl cinnamal, anthracene, benzophenone, benzothiazole, benzyl benzoate, benzyl salicylate, bis(2-ethylhexyl)phthalate, butyl benzyl phthalate, butylated hydroxyanisole, butylated hydroxytoluene, caffeine, coumarin, d-limonene, di-n-butyl phthalate, di-n-hexyl phthalate, di-n-nonyl phthalate, di-n-octyl phthalate, diethyl phthalate, diisobutyl phthalate, ethylene brassylate, eugenol, exaltolide (15-pentadecanolide), fluorene, galaxolide, hydroxy-citronellal, lilial, linalool, musk ketone, n,n-diethyl-m-toluamide, pyrene, thymol, tonalide, triclosan

Table S3. Venn diagram table for the 30% most commonly detected chemicals in three unique groups of wristbands, corresponding to Figure 3B. Black dots with a blue background represent the wristband groups included for each intersection of the Venn diagram.

Intersections	Wristband Groups: North America	Wristband Groups: South America	Wristband Groups: Africa	Chemicals in Common: Number	Chemicals in Common: Names
One	•			23	2,4-di-tert-butylphenol, 2-methylphenanthrene, 4-tert-butylphenol, PBDE 47, TCPP, benzothiazole, benzyl alcohol, bifenthrin, butylated hydroxyanisole, cinnamal, coumarin, di-n-hexyl phthalate, dicyclohexyl phthalate, dimethyl phthalate, ethofenprox, exaltolide (15-pentadecanolide), linalool, naphthalene, permethrin, permethrin II, tributyl phosphate, triclosan, tris(2-ethylhexyl) phosphate
		•		7	4,4'-DDE, 4-isopropylphenol, chlorpyrifos, diuron metabolite (3,4-dichlorophenyl isocyanate), guaiacol, isoeugenol, pyrene
			•	0	
Two		•	•	1	musk ketone
	•		•	0	
	•	•		12	1-methylnaphthalene, 4-chlorophenyl isocyanate, TPP, anthracene, b-citronellol, b-ionone, benzyl benzoate, butyl benzyl phthalate, caffeine, d-limonene, lilial, piperonyl butoxide
Three	•	•	•	13	amyl cinnamal, benzophenone, benzyl salicylate, bis(2-ethylhexyl)phthalate, butylated hydroxytoluene, di-n-butyl phthalate, di-n-nonyl phthalate, diethyl phthalate, diisobutyl phthalate, ethylene brassylate, galaxolide, n,n-diethyl-m-toluamide, tonalide

Table S4. Venn diagram table for all detected chemicals in five unique groups of wristbands, corresponding to Figure 3C. Black dots with a blue background represent the wristband groups included for each intersection of the Venn diagram.

Intersections	Wristband Groups: US Rural	US Urban	Peru Rural	Peru Urban	Senegal & South Africa	Chemicals in Common: Number	Names
One	●					6	2-sec-butylphenol, 9-methylanthracene, cyclopentadecanone, cyphenothrin trans-, fipronil desulfinyl-, tetrachlorvinphos
		●				48	1-naphthylamine, 2,3,6-trimethylphenol, 2,4-dimethylaniline, 2-chlorosyringaldehyde, 2-isopropylphenol, PBB 1, PBDE 51, PBDE 88, PBDE 100, PBDE 119, PBDE 49, PCB 102, PCB 88, PCB 93, PCB 95, PCB 98, acenaphthene, alpha-chlordane, benzo[a]fluoren-11-one, benzo[ghi]perylene, benzyl cinnamate, bioallethrin S-cyclopentenyl isomer, bisphenol A, citral B, cyfluthrin I, cyfluthrin II, cyfluthrin III, cypermethrin-3, d-(cis-trans)-phenothrin-I, gamma-chlordane, jasmolin II, kinoprene, methyleugenol, p-nitrotoluene, pentachloroanisole, phenoxyacetic acid, phthalimide, promecarb, pyriproxyfen, quinolone, rabenzazole, terbucarb, theobromine, tilt, trans-nonachlor, tri-p-tolyl phosphate, tricresylphosphate ortho-, triphenylene
			●			13	1,5-dimethylnaphthalene, 1-methylpyrene, 2,4'-DDT, 2-ethylnaphthalene, 4-chlorophenol, bendiocarb, benzo[a]pyrene, butachlor, cyclopenta[cd]pyrene, dichlorvos, diphenylamine, tebuconazole, trifloxystrobin
				●		3	3,5-dichloroaniline, pirimiphos-methyl, traseolide
					●	6	1-hydroxynaphthalene, 2,3,5-trimethylphenol, musk xylene, profenofos metabolite (4-bromo-2-chlorophenol), propoxur, tetramethrin I
Two				●	●	0	
			●		●	0	
			●	●		6	2,6-dimethoxyphenol, 4,4'-DDE, 4-methylphenol, 9-fluorenone, bisphenol AF, tetramethrin II
		●			●	2	cyhalothrin (gamma), cyhalothrin I (lambda)
		●		●		4	3-chloroaniline, 3-tert-butylphenol, citral A, diamyl phthalate
		●	●			11	1,2-dimethylnaphthalene, 1,6-dimethylnaphthalene, 4,4'-DDD, benz[a]anthracene, biphenyl, cashmeran, chrysene, farnesol IV, geraniol, phenanthrene, retene
	●				●	0	
	●			●		1	malathion
	●		●			2	2,3-dimethylanthracene, fluoranthene
	●	●				14	4-tert-butylphenol, PBDE 47, PBDE 99, benzo[a]fluorene, bifenthrin, carvone, cinnamal, drometrizole, ethofenprox, fipronil-sulfone, isobornyl thiocyanoacetate, methoprene II, tricresylphosphate meta-, tris(2-chloroethyl) phosphate
Three			●	●	●	0	
		●		●	●	0	
		●	●		●	0	
		●	●	●		7	2,4-dichlorophenol, 2-methylnaphthalene, 4,4'-DDT, acenaphthylene, diuron metabolite (3,4-dichlorophenyl isocyanate), isoeugenol, promecarb artifact (5-isopropyl-3-methylphenol)
	●			●	●	0	
	●		●		●	0	
	●		●	●		3	chlorpyrifos, cyphenothrin cis-, guaiacol
	●	●			●	3	benzyl alcohol, celestolide, triethyl phosphate
	●	●		●		3	dimethyl phthalate, lyral, o-phenylphenol
	●	●	●			11	1-methylphenanthrene, 2-methylphenanthrene, 3,6-dimethylphenanthrene, 4-chloro-3,5-dimethylphenol, benzo[b]fluorene, fipronil, naphthalene, nicotine, permethrin, permethrin II, tributyl phosphate
Four	●	●	●	●		12	2,4-di-tert-butylphenol, 2-methoxy-4-methylphenol, 4-isopropylphenol, a-ionone, b-citronellol, b-ionone, cypermethrin-2, dicyclohexyl phthalate, piperonyl butoxide, triphenyl phosphate, tris(1-chloro-2-propyl) phosphate, tris(2-ethylhexyl) phosphate
	●	●	●		●	4	2,6-dimethylnaphthalene, benzothiazole, butylated hydroxyanisole, hydroxy-citronellal
	●	●		●	●	1	di-n-hexyl phthalate
	●		●	●	●	0	
		●	●	●	●	3	eugenol, fluorene, thymol
Five	●	●	●	●	●	28	1-methylnaphthalene, 4-chlorophenyl isocyanate, amyl cinnamal, anthracene, benzophenone, benzyl benzoate, benzyl salicylate, bis(2-ethylhexyl)phthalate, butyl benzyl phthalate, butylated hydroxytoluene, caffeine, coumarin, d-limonene, di-n-butyl phthalate, di-n-nonyl phthalate, di-n-octyl phthalate, diethyl phthalate, diisobutyl phthalate, ethylene brassylate, exaltolide (15-pentadecanolide), galaxolide, lilial, linalool, musk ketone, n,n-diethyl-m-toluamide, pyrene, tonalide, triclosan

Table S5. Venn diagram table for the 30% most commonly detected chemicals in five unique groups of wristbands, corresponding to Figure 3D. Black dots with a blue background represent the wristband groups included for each intersection of the Venn diagram.

Intersections	Wristband Groups					Chemicals in Common	
	US Rural	US Urban	Peru Rural	Peru Urban	Senegal & South Africa	Number	Names
One	•					2	di-n-hexyl phthalate, tris(2-ethylhexyl) phosphate
		•				17	2,4-di-tert-butylphenol, 4-chlorophenyl isocyanate, 4-tert-butylphenol, PBB 1, PBDE 47, benzothiazole, benzyl alcohol, butylated hydroxyanisole, cinnamal, d-limonene, dicyclohexyl phthalate, dimethyl phthalate, ethofenprox, linalool, naphthalene, permethrin II, tributyl phosphate
			•			8	4,4'-DDE, 4-isopropylphenol, chlorpyrifos, exaltolide (15-pentadecanolide), guaiacol, isoeugenol, pyrene, trifloxystrobin
				•		1	diuron metabolite (3,4-dichlorophenyl isocyanate)
					•	0	
Two				•	•	1	musk ketone
			•		•	0	
			•	•		0	
		•			•	0	
		•		•		0	
		•	•			3	1-methylnaphthalene, 2-methylphenanthrene, coumarin
	•				•	0	
	•			•		0	
	•		•			0	
	•	•				4	b-ionone, permethrin, triclosan, tris(1-chloro-2-propyl) phosphate
Three			•	•	•	0	
		•		•	•	0	
		•	•		•	0	
		•	•	•		0	
	•			•	•	0	
	•		•		•	0	
	•		•	•		0	
	•	•			•	0	
	•	•		•		1	b-citronellol
	•	•	•			2	benzyl benzoate, piperonyl butoxide
Four	•	•	•	•		5	anthracene, butyl benzyl phthalate, caffeine, lilial, triphenyl phosphate
	•	•	•		•	0	
	•	•		•	•	0	
	•		•	•	•	0	
		•	•	•	•	0	
Five	•	•	•	•	•	13	amyl cinnamal, benzophenone, benzyl salicylate, bis(2-ethylhexyl)phthalate, butylated hydroxytoluene, di-n-butyl phthalate, di-n-nonyl phthalate, diethyl phthalate, diisobutyl phthalate, ethylene brassylate, galaxolide, n,n-diethyl-m-toluamide, tonalide

Table S6. Tukey-Kramer HSD results for different regions, represented by (A) connecting letters of significance and (B) p-values. For the connecting levels, levels not connected by the same letter are significantly different. Wristband groups highlighted in grey are not included because of a small sample size.

A

Wristband Group	Connecting Letters						Mean Chemicals Detected
Total Chemicals Detected							
Texas	A						28.2
North Carolina	A	B					24.6
Oregon		B					23.9
New York		B	C				21.3
South Africa							19.0
Alto Mayo				D			17.8
Washington, D.C.			C	D			17.7
Ohio			C	D			17.6
Senegal					E		10.0
Potential Endocrine Disrupting Chemicals Detected							
Texas	A						19.0
North Carolina		B	C				16.2
Oregon	A	B					16.9
New York			C	D			14.0
South Africa							12.5
Alto Mayo				D			13.6
Washington, D.C.				D			12.9
Ohio				D			13.3
Senegal					E		7.8

B

Comparing Two Wristband Groups		Tukey-Kramer HSD p-value	
**Group 1**	**Group 2**	**Total Chemicals Detected**	**Potential Endocrine Disrupting Chemicals Detected**
Texas	Senegal	**<0.0001***	**<0.0001***
Texas	Ohio	**<0.0001***	**<0.0001***
Texas	Washington, D.C.	**<0.0001***	**<0.0001***
Texas	Alto Mayo	**<0.0001***	**<0.0001***
Texas	New York	**<0.0001***	**<0.0001***
Texas	Oregon	**0.01***	0.11
Texas	North Carolina	0.17	**0.0496***
Oregon	Senegal	**<0.0001***	**<0.0001***
Oregon	Ohio	**<0.0001***	**0.0002***
Oregon	Washington, D.C.	**<0.0001***	**<0.0001***
Oregon	Alto Mayo	**<0.0001***	**<0.0001***
Oregon	New York	0.44	**0.007***
Ohio	Senegal	**<0.0001***	**<0.0001***
North Carolina	Senegal	**<0.0001***	**<0.0001***
North Carolina	Ohio	**<0.0001***	**0.04***
North Carolina	Washington, D.C.	**<0.0001***	**0.01***
North Carolina	Alto Mayo	**<0.0001***	**0.02***
North Carolina	New York	0.32	0.25

North Carolina	Oregon	1.00	0.99
New York	Senegal	**<0.0001***	**<0.0001***
New York	Ohio	0.14	0.99
New York	Washington, D.C.	0.17	0.95
New York	Alto Mayo	**0.047***	1.00
Washington, D.C.	Senegal	**<0.0001***	**<0.0001***
Washington, D.C.	Ohio	1.00	1.00
Alto Mayo	Senegal	**<0.0001***	**<0.0001***
Alto Mayo	Ohio	1.00	1.00
Alto Mayo	Washington, D.C.	1.00	0.98

Table S7. Tukey-Kramer HSD results for rural and urban groups within different continents, represented by (A) connecting letters of significance and (B) p-values. For the connecting levels, levels not connected by the same letter are significantly different. Wristband groups highlighted in grey are not included because of a small sample size.

A

Wristband Group	Connecting Letters						Mean Chemicals Detected
Total Chemicals Detected							
North America Urban	A						23.1
North America Rural		B					19.9
Africa Urban							19.0
South America Urban		B					19.0
South America Rural		B					17.3
Africa Rural			C				10.0
Potential Endocrine Disrupting Chemicals Detected							
North America Urban	A						15.9
North America Rural	A	B					14.6
Africa Urban							12.5
South America Urban	A	B					14.2
South America Rural		B					13.4
Africa Rural			C				7.8

B

Comparing Two Wristband Groups		Tukey-Kramer HSD p-value	
**Group 1**	**Group 2**	**Total Chemicals Detected**	**Potential Endocrine Disrupting Chemicals Detected**
North America Urban	Africa Rural	**<0.0001***	**<0.0001***
North America Rural	Africa Rural	**<0.0001***	**<0.0001***
South America Urban	Africa Rural	**<0.0001***	**<0.0001***
South America Rural	Africa Rural	**<0.0001***	**<0.0001***
North America Urban	South America Rural	**<0.0001***	**0.0004***
North America Urban	South America Urban	**0.02***	0.25
North America Rural	South America Rural	0.21	0.59
North America Urban	North America Rural	**0.02***	0.30
North America Rural	South America Urban	0.98	0.99
South America Urban	South America Rural	0.77	0.93

Table S8. Tukey-Kramer HSD results for different age groups, represented by (A) connecting letters of significance and (B) p-values. For the connecting levels, levels not connected by the same letter are significantly different. Wristband groups highlighted in grey are not included because of a small sample size.

A

Wristband Group	Connecting Letters						Mean Chemicals Detected
Total Chemicals Detected							
North America >60	A						29.1
North America <11	A	B					25.1
North America 41-60	A	B	C				24.2
North America 21-40	A	B	C				22.2
South America >60		B	C	D			20.6
South America <11	A	B	C	D	E		20.0
South America 11-20			C	D	E		17.2
South America 41-60				D	E		17.0
South America 21-40				D	E		17.1
Africa 11-20							10.0
Africa 21-40						F	10.0
Africa 41-60					E	F	9.8
Potential Endocrine Disrupting Chemicals Detected							
North America >60	A						21.4
North America <11	A	B					17.4
North America 41-60	A	B	C				17.2
North America 21-40			C	D			14.5
South America >60		B	C	D			15.8
South America <11	A	B	C	D			15.3
South America 11-20		B	C	D	E		13.7
South America 41-60				D	E		12.8
South America 21-40				D			13.2
Africa 11-20							8.0
Africa 21-40						F	7.6
Africa 41-60					E	F	7.5

B

Comparing Two Wristband Groups		Tukey-Kramer HSD p-value	
**Group 1**	**Group 2**	**Total Chemicals Detected**	**Potential Endocrine Disrupting Chemicals Detected**
North America >60	Africa 41-60	**<0.0001***	**<0.0001***
North America >60	Africa 21-40	**<0.0001***	**<0.0001***
North America <11	Africa 41-60	**<0.0001***	**<0.0001***
North America <11	Africa 21-40	**<0.0001***	**<0.0001***
North America 41-60	Africa 41-60	**<0.0001***	**<0.0001***
North America 41-60	Africa 21-40	**<0.0001***	**<0.0001***
North America 21-40	Africa 41-60	**0.0006***	**0.003***
North America 21-40	Africa 21-40	**<0.0001***	**<0.0001***
North America >60	South America 41-60	**<0.0001***	**<0.0001***
North America >60	South America 21-40	**<0.0001***	**<0.0001***
North America >60	South America 11-20	**0.0005***	**0.0002***
South America >60	Africa 41-60	**0.03***	**0.001***

South America >60	Africa 21-40	**0.0001***	**<0.0001***
South America <11	Africa 41-60	0.17	**0.03***
South America <11	Africa 21-40	**0.03***	**0.002***
North America >60	South America <11	0.16	0.08
North America >60	South America >60	**0.047***	**0.02***
North America <11	South America 41-60	**<0.0001***	**<0.0001***
North America <11	South America 21-40	**<0.0001***	**0.0001***
North America <11	South America 11-20	**0.003***	0.07
South America 11-20	Africa 41-60	0.37	0.06
South America 21-40	Africa 41-60	0.26	**0.047***
South America 41-60	Africa 41-60	0.26	0.08
South America 11-20	Africa 21-40	**0.04***	**0.0005***
North America 41-60	South America 41-60	**0.002***	**0.003***
North America 41-60	South America 21-40	**0.003***	**0.01***
South America 21-40	Africa 21-40	**0.002***	**<0.0001***
South America 41-60	Africa 21-40	**0.002***	**<0.0001***
North America >60	North America 21-40	0.053	**<0.0001***
North America 41-60	South America 11-20	0.06	0.25
North America 21-40	South America 41-60	**0.01***	0.73
North America <11	South America <11	0.74	0.97
North America 21-40	South America 21-40	**0.02***	0.95
North America >60	North America 41-60	0.59	0.14
North America 21-40	South America 11-20	0.30	1.00
North America <11	South America >60	0.39	0.95
North America 41-60	South America <11	0.94	0.99
North America >60	North America <11	0.70	0.08
North America 41-60	South America >60	0.85	0.99
South America >60	South America 41-60	0.82	0.41
South America >60	South America 21-40	0.84	0.64
South America >60	South America 11-20	0.96	0.95
South America <11	South America 41-60	0.99	0.95
North America <11	North America 21-40	0.37	0.99
South America <11	South America 21-40	0.99	**0.006***
South America <11	South America 11-20	1.00	1.00
North America 21-40	South America <11	1.00	1.00
North America 41-60	North America 21-40	0.97	0.19
North America 21-40	South America >60	1.00	1.00
North America <11	North America 41-60	1.00	1.00
South America >60	South America <11	1.00	1.00
Africa 21-40	Africa 41-60	1.00	1.00
South America 11-20	South America 41-60	1.00	1.00
South America 11-20	South America 21-40	1.00	1.00
South America 21-40	South America 41-60	1.00	1.00

Table S9. Tukey-Kramer HSD results for males and females within different continents, represented by (A) connecting letters of significance and (B) p-values. For the connecting levels, levels <u>not</u> connected by the same letter are significantly different.

A

Wristband Group	Connecting Letters						Mean Chemicals Detected
Total Chemicals Detected							
North America Male	A						24.5
North America Female	A	B					21.7
South America Female		B	C				18.6
South America Male			C				16.8
Africa Female			C	D			12.3
Africa Male				D			10.0
Potential Endocrine Disrupting Chemicals Detected							
North America Male	A						16.7
North America Female		B					14.8
South America Female	A	B	C				14.7
South America Male			C	D			12.5
Africa Female				D	E		9.5
Africa Male					E		7.6

B

Comparing Two Wristband Groups		Tukey-Kramer HSD p-value	
**Group 1**	**Group 2**	**Total Chemicals Detected**	**Potential Endocrine Disrupting Chemicals Detected**
South America Male	Africa Male	**0.0001***	**<0.0001***
South America Male	Africa Female	0.61	0.51
South America Female	South America Male	0.74	0.07
South America Female	Africa Male	**<0.0001***	**<0.0001***
South America Female	Africa Female	0.23	**0.04***
North America Male	South America Male	**<0.0001***	**<0.0001***
North America Male	South America Female	**<0.0001***	0.07
North America Male	North America Female	0.07	**0.04***
North America Male	Africa Male	**<0.0001***	**<0.0001***
North America Male	Africa Female	**0.0003***	**0.0005***
North America Female	South America Male	**0.0004***	**0.01***
North America Female	South America Female	0.07	1.00
North America Female	Africa Male	**<0.0001***	**<0.0001***
North America Female	Africa Female	**0.01***	**0.02***
Africa Female	Africa Male	0.97	0.90

Figure S1. Chemical pairs detected together ≥20 times in wristband samples. Sixteen chemicals are included in these pairs: piperonyl butoxide, butylated hydroxyanisole, PBDE 47, 2-methylphenanthrene, triphenyl phosphate (TPP), permethrin, dimethyl phthalate, naphthalene, benzyl alcohol, benzothiazole, 2,4-di-tert-butylphenol, cinnamal, b-ionone, b-citronellol, tris(1-chloro-2-propyl) phosphate (TCPP), and permethrin II. Each dot represents one of the 191 unique chemicals detected in this study. The width of the line corresponds to the number of wristbands with that chemical pair.

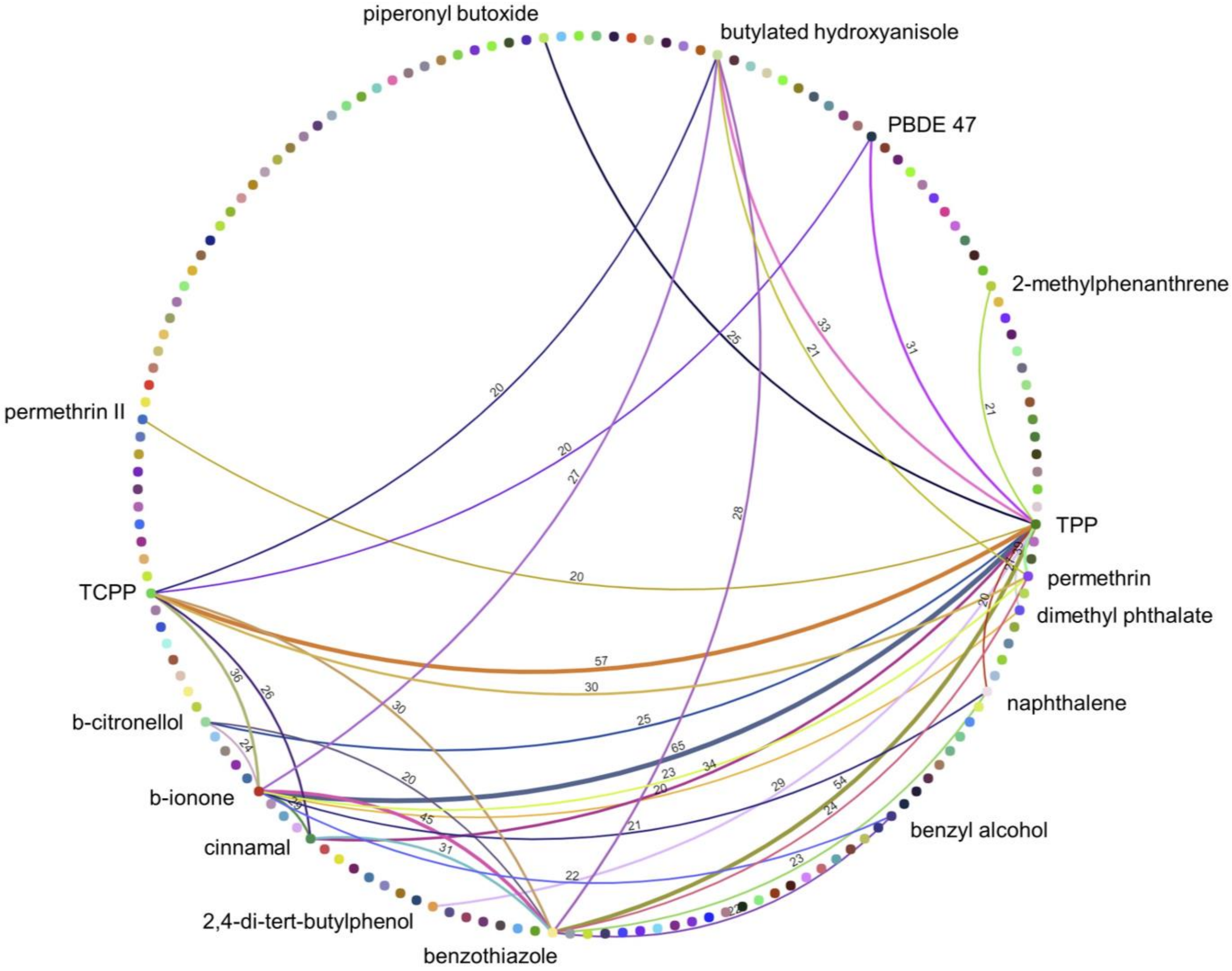

Figure S2. The PCA shown in Figure 5, but the symbol color here indicates the presence (orange) and absence (pink) of bis(2-ethylhexyl)phthalate. The black arrow indicates the direction of increasing density of presence for bis(2-ethylhexyl)phthalate.

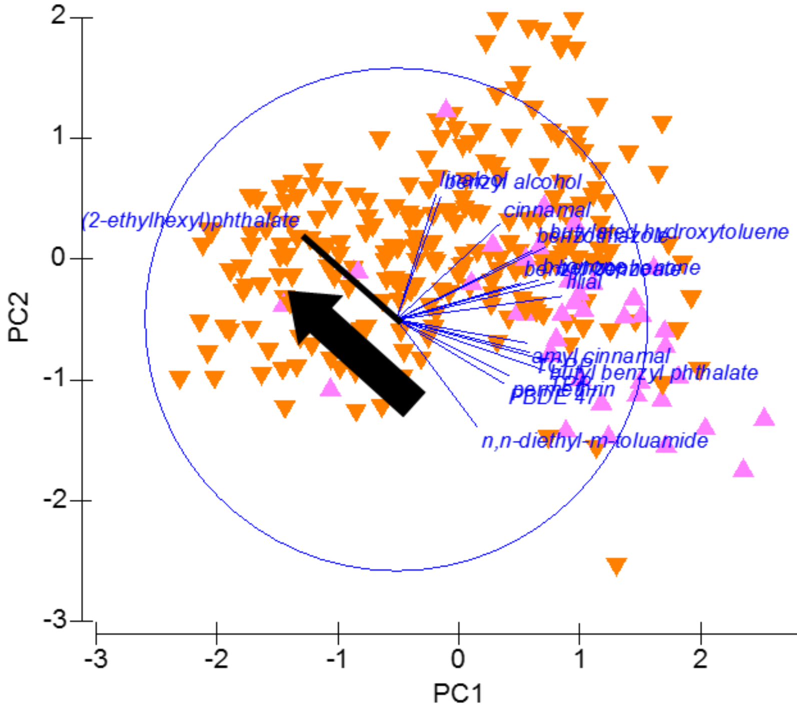

Figure S3. Number of chemical detections compared with wristband deployment time for all wristbands.

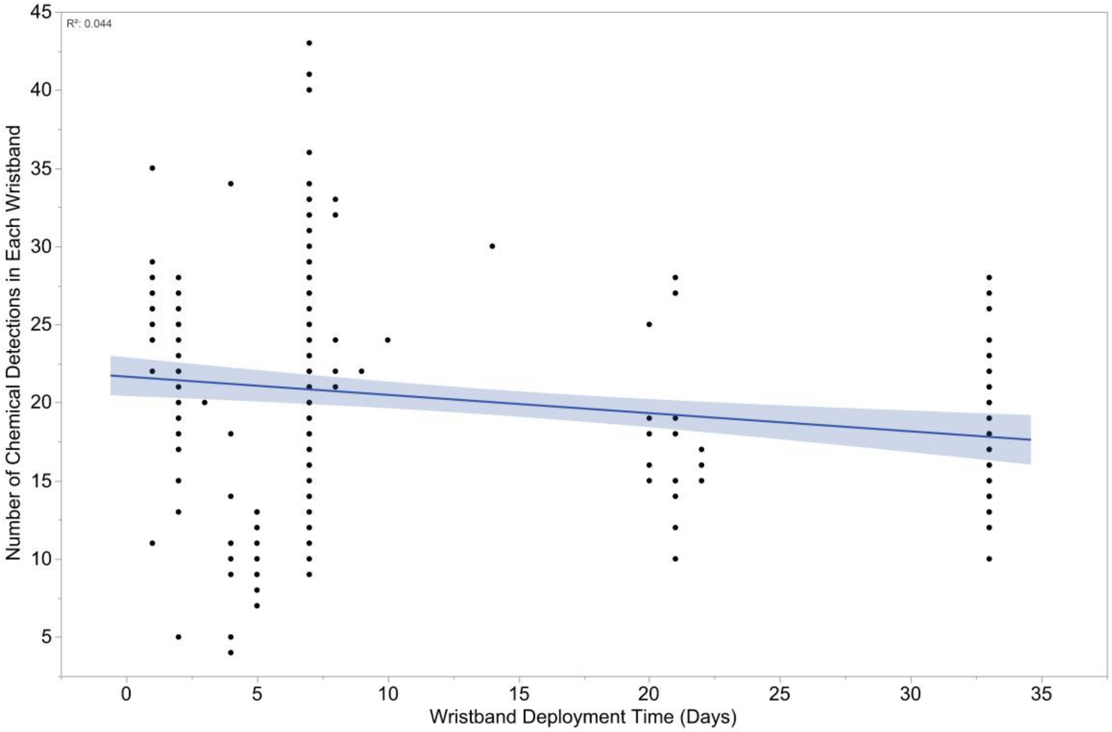

References

1 Bergmann, A. J., Points, G. L., Scott, R. P., Wilson, G., Anderson, K. A. 2018 Development of quantitative screen for 1550 chemicals with GC-MS. *Anal. Bioanal. Chem.* **410**, 3101-3110. (10.1007/s00216-018-0997-7)
